# Evaluation of Cholinergic Deficiency in Preclinical Alzheimer's Disease Using Pupillometry

**DOI:** 10.1155/2017/7935406

**Published:** 2017-08-16

**Authors:** Shaun Frost, Liam Robinson, Christopher C. Rowe, David Ames, Colin L. Masters, Kevin Taddei, Stephanie R. Rainey-Smith, Ralph N. Martins, Yogesan Kanagasingam

**Affiliations:** ^1^Commonwealth Scientific and Industrial Research Organisation (CSIRO), Perth, WA, Australia; ^2^Australian e-Health Research Centre, Perth, WA, Australia; ^3^Department of Nuclear Medicine and Centre for PET, Austin Health, Melbourne, VIC, Australia; ^4^Department of Psychiatry, University of Melbourne, Melbourne, VIC, Australia; ^5^The Mental Health Research Institute (MHRI), University of Melbourne, Melbourne, VIC, Australia; ^6^National Ageing Research Institute, Melbourne, VIC, Australia; ^7^School of Medical and Health Sciences, Edith Cowan University, Joondalup, WA, Australia; ^8^Sir James McCusker Alzheimer's Disease Research Unit, Hollywood Private Hospital, Perth, WA, Australia; ^9^School of Biomedical Sciences, Macquarie University, North Ryde, NSW, Australia; ^10^School of Psychiatry and Clinical Neurosciences, University of Western Australia, Crawley, WA, Australia

## Abstract

Cortical cholinergic deficiency is prominent in Alzheimer's disease (AD), and published findings of diminished pupil flash response in AD suggest that this deficiency may extend to the visual cortical areas and anterior eye. Pupillometry is a low-cost, noninvasive technique that may be useful for monitoring cholinergic deficits which generally lead to memory and cognitive disorders. The aim of the study was to evaluate pupillometry for early detection of AD by comparing the pupil flash response (PFR) in AD (*N* = 14) and cognitively normal healthy control (HC, *N* = 115) participants, with the HC group stratified according to high (*N* = 38) and low (*N* = 77) neocortical amyloid burden (NAB). Constriction phase PFR parameters were significantly reduced in AD compared to HC (maximum acceleration *p* < 0.05, maximum velocity *p* < 0.0005, average velocity *p* < 0.005, and constriction amplitude *p* < 0.00005). The high-NAB HC subgroup had reduced PFR response cross-sectionally, and also a greater decline longitudinally, compared to the low-NAB subgroup, suggesting changes to pupil response in preclinical AD. The results suggest that PFR changes may occur in the preclinical phase of AD. Hence, pupillometry has a potential as an adjunct for noninvasive, cost-effective screening for preclinical AD.

## 1. Introduction

**Figure 1:**
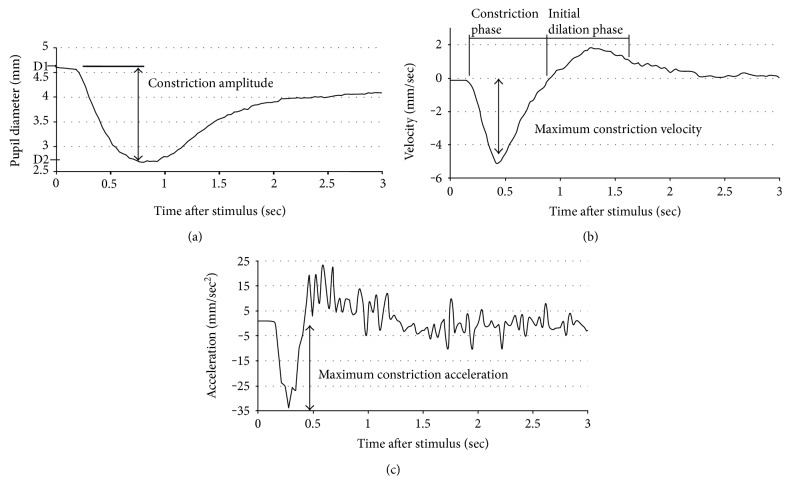
Illustration of pupil flash response and parameters measured. (a) Pupil diameter over time after stimulus at time zero. (b) Pupil velocity (rate of change of pupil diameter). (c) Pupil acceleration (rate of change of velocity). The constriction phase lasts from stimulus to minimum pupil size; parameters calculated during this phase are the constriction amplitude, maximum and average constriction velocity, and maximum constriction acceleration.

The ocular pupil controls retinal illumination and responds dynamically to a bright flash of light by rapid constriction followed by redilation (Figure 1). Pupillometry investigates this response by delivering a flash of light into the eye and accurately detecting and measuring pupil size changes over time.

Pupil size and response are controlled by the opposing action of the sphincter and dilator muscles of the iris. The constriction phase of the pupil response (Figure 1) is primarily driven by the cholinergic system [1], with acetylcholine (ACh) being the neurotransmitter involved in projections between the Edinger-Westphal nucleus, ciliary ganglion, and sphincter muscle [2]. Thus, pupillometry provides a practical, noninvasive approach with which to evaluate cholinergic deficiency.

Pupillometry has been used to identify a cholinergic deficiency in a number of disorders including Alzheimer's disease (AD) [3–8] and Parkinson's disease [5, 9]. As the primary neurotransmitter deficit in AD is ACh [10–12], the constriction phase of the pupil flash response (PFR) has gained interest for evaluating cholinergic deficiency and for early detection, diagnosis, and monitoring of AD. The majority of study results to date indicate a slower and reduced pupil response in AD, with reduced velocities, accelerations, and constriction amplitude, and increased latencies. The work of Ferrario et al. [6] on only constriction acceleration stands in contrast, possibly due to different methodology and participant selection. Some studies indicate a faster recovery after stimulus in AD, despite slower constriction and dilation velocities, probably due to the reduced amplitude [3, 13].

Our prior study [8] found a weaker pupil constriction response in AD, consistent with the hypothesis of a cholinergic deficit in the peripheral parasympathetic pathway in AD. Significant differences were found between AD and cognitively normal healthy control (HC) participants in 10 different calculated PFR parameters, with the greatest differences coming from the constriction phase: maximum constriction acceleration, maximum constriction velocity, mean constriction velocity, and constriction amplitude. These promising preliminary results warranted further investigation into whether pupil response changes occur early in AD, possibly providing a test for early detection or monitoring of the disease.

AD is characterized clinically by a progressive decline in memory, learning, and executive function and neuropathologically by the presence of cerebral extracellular amyloid deposits (plaques), intracellular neurofibrillary tangles, and cerebral (in particular hippocampal) atrophy. In addition to the debilitating symptoms endured by AD patients, the disease imposes a huge social and economic burden on society [14].

AD cognitive symptoms arise only after extensive, irreversible neural deterioration has already occurred. As a result, diagnosis is usually made late in the disease process, limiting both the efficacy of available treatments and the evaluation of new treatments. Biomarkers for early detection of AD include cerebrospinal fluid concentrations of beta-amyloid (A*β*), total tau and phosphorylated tau peptides [15–18], and brain A*β* plaque burden imaged using positron emission tomography (PET) [16, 18–20]. Research demonstrates that plaque burden can be detected over 20 years before cognitive symptoms begin [21]. However, while these are valuable diagnostic and secondary screening biomarkers, they are not suitable as primary screening technologies for AD. A screening process that could provide early, accurate diagnosis or a prognosis of AD would enable earlier intervention, facilitate cost-effective screening into treatment trials, and allow current and future treatments to be more effective.

The present study investigated constriction phase pupil response in AD and HC participants, with a particular focus on PFR changes in preclinical AD as determined by high neocortical amyloid burden (NAB).

## 2. Materials and Methods

### 2.1. Participants

Participants for the study were recruited from the Australian Imaging, Biomarkers, and Lifestyle (AIBL) study of ageing in Australia. The AIBL study has two sites: Melbourne (Victoria) and Perth (Western Australia). The pupillometry study was conducted only at the Perth site. A previous report details the AIBL study design and baseline cohort [22]. Briefly, the AIBL study integrates data from neuroimaging, biomarkers, lifestyle, clinical, and neuropsychological domains for eligible volunteers older than 60 years who are fluent in English. AIBL classifies participants into 3 groups: (1) individuals meeting the criteria for AD based on the NINCDS-ADRDA (National Institute of Neurological and Communicative Disorders and Stroke-Alzheimer's Disease and Related Disorders Association) [23], (2) individuals meeting the criteria for mild cognitive impairment [24, 25], and (3) cognitively normal healthy control individuals.

The following were part of the AIBL exclusion criteria: a history of non-AD dementia, schizophrenia, bipolar disorder, significant current (but not past) depression (geriatric depression scale above 5/15), Parkinson's disease, cancer (other than basal cell skin carcinoma) within the last 2 years, symptomatic stroke, uncontrolled diabetes, diagnosed obstructive sleep apnoea, or current regular alcohol use exceeding 2 standard drinks per day for women or 4 per day for men.

AIBL participants were excluded from the pupil response study if they did not have PET data available; if they had pupillary malformations, severe cataract, self-reported history of glaucoma in either eye, penetrating eye wounds to both eyes, and eye surgery to both eyes that involved the muscle; if they used cholinesterase inhibitors or prescribed ocular medications; or if they were unable to complete the task without excessive blinking.

All participants provided written informed consent, and all PFR procedures were approved by the Hollywood Private Hospital Research Ethics Committee according to the Helsinki Declaration. Approval for the parent AIBL study was obtained from the Austin Health Human Research Ethics Committee, the Hollywood Private Hospital Research Ethics Committee, the St. Vincent Hospital Research Ethics Committee, and the Edith Cowan University Human Research Ethics Committee.

The present study draws upon data generated from the AIBL study, including neuroimaging and genetic test results. The methodology for these AIBL procedures is reported elsewhere [22] and summarized below.

### 2.2. Neuroimaging

Neuroimaging methodology is reported in more detail elsewhere [26]. Briefly, participants were neuroimaged for the presence of fibrillar brain A*β* using PET with 2 different radiotracers: 11C-Pittsburgh compound B (PiB) and 18F-flutemetamol (FLUTE). Previous reports describe the PET methodology for each tracer in detail [20, 27]. For semiquantitative analysis, a volume of interest template was applied to the summed and spatially normalized PET images to obtain a standardized uptake value (SUV). The images were then scaled to the SUV of each tracer's recommended reference region to generate a tissue ratio termed SUV ratio (SUVR). A global measure of NAB was computed using the mean SUVR in the frontal, superior parietal, lateral temporal, occipital, and anterior and posterior cingulate regions of the brain. For PiB, the SUV was normalized to the cerebellar cortex, whereas the pons was used as the reference region for FLUTE [28]. SUVR was stratified into a dichotomous variable classified as high or low based on neuropathologically validated thresholds for each tracer. We considered participants who underwent FLUTE imaging to have high NAB when the SUVR was 0.62 or higher [28], and for PiB imaging, when the SUVR was 1.4 or higher [29].

### 2.3. Genotyping

APOE genotyping was performed according to the following protocol: fasting blood samples were obtained using standard venepuncture of the antecubital vein and collected into EDTA tubes containing prostaglandin E1 (PGE: 33.3 ng/ml; Sapphire Bioscience, NSW, Australia) to prevent platelet activation. Extraction of DNA from 5 ml of whole blood was undertaken using QIAamp DNA Blood Maxi Kits (Qiagen, Hilden, Germany) as per the manufacturer's instructions. Specific TaqMan® (Thermo Fisher Scientific, Waltham, MA, USA) genotyping assays were used for ascertaining APOE genotype (rs7412, assay ID: C____904973_10; rs429358, assay ID: C___3084793_20), which were performed on a QuantStudio 12K Flex™ real-time PCR system (Thermo Fisher Scientific, Waltham, MA, USA) using the TaqMan GTXpress™ Master Mix (Thermo Fisher Scientific, Waltham, MA, USA).

### 2.4. System of Pupillometry

A record of the pupil's response to a flash of light was collected for each participant using a commercial pupillometer. The PFR was collected using a NeurOptics™ VIP™-200 Pupillometer. This is a commercial, monocular device providing fully automated operation and calculation of response parameters. The device produces a white flash stimulus and then measures the pupil size for 5 seconds using infrared illumination. The video frame rate is 33 Hz, the stimulus/pulse intensity is 180 *μ*W, and the stimulus/pulse duration is 31 ms. The pupillometer produces diffuse light over the whole visual field.

The room was darkened for 2 minutes prior to testing. The test was practiced once before recording. Occasionally, an extra trial was needed to achieve a recording without blinks or artefacts. Data was rejected if artefacts were present. The right eye was used for all participants, except where there was injury or pathology to the right eye only or a suitable pupil response could not be obtained with the right eye, in which case the left eye was used (*N* = 1). The pupillometer provided automatic calculation of the following pupil response parameters: resting pupil diameter (D1, mm), minimum pupil diameter (D2, mm), average constriction velocity (CV, mm/sec), maximum constriction velocity (MCV, mm/sec), and constriction amplitude (AMP, mm), which was calculated as the difference between resting pupil diameter and minimum pupil diameter (D1 − D2, mm). A record of the pupil's diameter as a function of time was exported from the pupillometer. From this record, maximum constriction acceleration (MCA, mm/sec^2^) was calculated by masked operators using fully automated computer algorithms. PFR trials with artefacts or excessive blinking were discarded. A computer algorithm was used to remove minor blinks.

### 2.5. Statistical Analysis

Descriptive statistics including means and standard deviations (SD) for the full cohort and clinical group are shown in Table 1. Demographic comparisons were performed using a *χ*^2^ test (Fisher's exact calculation where necessary) for categorical variables (gender and *APOE ε*4 status), and analysis of variance (ANOVA) for the continuous age variable (*p* < 0.05 considered significant). Pupil response measures were compared between groups using generalised linear modelling, with confounding variables reduced via the stepAIC method (a stepwise model selection by Akaike information criterion). Confounders considered included age, sex, and *APOE ε*4 status (the major genetic risk factor for sporadic AD [30]). Statistical significance was defined as *p* < 0.05. All statistical analyses were conducted in the R statistical environment [31]. The likelihood of false-positive results was minimised by comparing *p* values to adjusted critical values according to the Benjamini and Hochberg false discovery rate (FDR) method [32]. Receiver-operating characteristic (ROC) curve analysis was also performed to further illustrate the classification accuracy of the PFR parameters. The area under the curve (AUC) of the ROC curves was estimated, an AUC of 1 indicates perfect classification ability, whereas an AUC near 0.5 indicates poor (random) classification ability. Logistical models combining PFR measures were created to assess combined classification performance.

## 3. Results

Eligible AIBL participants in Perth with PET data available numbered 206 (182 HC, 24 AD). *N* = 180 (87%) were willing to participate in the pupillometry study. Participants were excluded from the pupil response study if they had pupillary malformations, severe cataract (*N* = 5), self-reported history of glaucoma in either eye, penetrating eye wounds to both eyes, and eye surgery to both eyes that involved the muscle; if they used cholinesterase inhibitors or prescribed ocular medications (*N* = 36); or if they were unable to complete the task without excessive blinking (*N* = 10). All participants were white Caucasians.

The pupillometry study thus included *N* = 129 participants (115 HC, 14 AD). Table 1 shows the demographic comparisons between HC and AD groups, Table 2 shows the same for the HC group stratified according to NAB status, and Table 3 shows the same for the 37 HC participants with longitudinal pupillometry results available, again stratified according to NAB status.

There was a significantly greater proportion of *APOE ε*4 carriers in the AD group (Table 1, *p* = 0.000001), consistent with *APOE ε*4 being the major genetic risk factor for sporadic AD. The AD group was also older (mean age 77.4 years) compared to the HC group (mean age 72.9 years) (*p* = 0.002).

PFR parameters were not significantly different between males and females, or between *APOE ε*4 carriers and noncarriers, but they did exhibit an age dependence (MCV *p* = 0.00002, CV *p* = 0.00001, MCA *p* = 0.002, and AMP *p* = 0.02).

**Figure 2:**
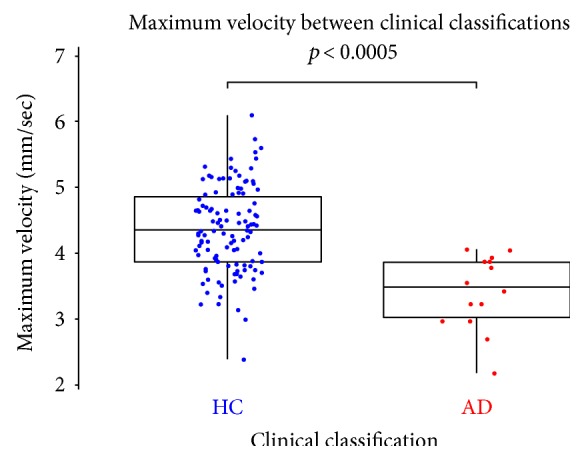
Comparison across clinical groups of the maximum velocity in the constriction phase of the pupil flash response. HC refers to healthy control participants, AD refers to Alzheimer's disease, mm refers to millimetres, and sec refers to seconds.

Significant differences in pupil response were found between the AD and HC groups (Table 1). Specifically, the AD group exhibited reduced MCV (*p* = 0.00045, Figure 2), AMP (*p* = 0.0030), MCA (*p* = 0.030), and CV (*p* = 0.0015). All results were significant after adjustment using the Benjamini and Hochberg FDR method [32].

MCV provided the greatest clinical classification accuracy with sensitivity 100%, specificity 67%, and AUC 0.85 (CI [0.76–0.93]). Combining PFR parameters into a logistic model did not improve classification performance, as the parameters were highly correlated. However, adding age and *APOE* є4 carrier status improved classification performance to sensitivity 91.7%, specificity 93.1%, and AUC 0.94 (CI [0.87–1]).

Stratifying the HC group according to NAB, the low-NAB group consisted of 77 participants of mean age 72.3 years, while the high-NAB group consisted of 38 participants of mean age 74.0 years. Demographics and results of this comparison are presented in Table 2. There was a significantly greater proportion of *APOE ε*4 carriers in the high-NAB group (*p* = 0.0002), again consistent with *APOE ε*4 being the major genetic risk factor for sporadic AD. MCV was reduced in the high-NAB group (*p* = 0.021). The remaining PFR parameters exhibited nonsignificant trends for reduced values in the high-NAB group. Combining MCV and MCA into a logistic model provided classification accuracy for high NAB with sensitivity 57.1%, specificity 71.6%, and AUC 0.63 (CI [0.52–0.75]). Adding age and *APOE ε*4 carrier status improved the performance of the model to sensitivity 60%, specificity 88%, and AUC 0.74 (CI [0.63–0.84]).

Thirty HC participants (19 low NAB, 11 high NAB) underwent longitudinal pupillometry, with PFR data collected using the same device and an intermeasurement period ranging 27–36 months prior to this study [8]. The change in PFR parameters between visits was calculated. Demographics and results of this comparison are presented in Table 3. There was again a significantly greater proportion of *APOE ε*4 carriers in the high-NAB group (*p* = 0.035). The high-NAB group also had a greater percentage of males (73%) compared to the low-NAB group (37%) (*p* = 0.00026). PFR parameters were not significantly correlated with the exact interval between longitudinal measurements.

Group means for each PFR parameter change were negative, indicating a weaker PFR at the more recent pupillometry test. The reduction in MCA and MCV was more pronounced in the high-NAB group (*p* = 0.0068, *p* = 0.047, resp.). The MCA result was still significant after Benjamini and Hochberg FDR adjustment [32]; however, the maximum velocity result was not. Combining MCA and MCV in a logistic model provided a classification accuracy for high NAB with sensitivity 73%, specificity 100%, and AUC 0.90 (CI [0.75–1]). Adding age and *APOE ε*4 carrier status improved the performance of the model to sensitivity 91%, specificity 100%, and AUC 0.99 (CI [0.96–1.0]).

## 4. Discussion

The results are indicative of a weaker constriction phase pupil response in AD, consistent with earlier studies [3–5, 9]. Possible causes of the PFR changes in AD are degeneration in relays in the midbrain or central cholinergic depletion [11, 12]. The four PFR parameters considered in this study are all measures of the constriction phase of the PFR which is primarily a parasympathetic cholinergic response [1]. These four PFR parameters were also the same parameters that were most significantly altered in AD in prior studies [3–8]. The results therefore suggest cholinergic deficits in the peripheral parasympathetic pathway in AD. AD patients receiving pharmacological treatment with anticholinesterase agents (such as Donepezil) have been excluded from this study, due to the likely effect of these drugs on the PFR. The necessary exclusion of those on anticholinesterase agents introduces some bias as it is possible that those not so treated are going to be different in some way from the 60–70% of AD subjects who do receive such therapy; for example, reported results from other studies indicate that Donepezil may normalize PFR in some AD patients [4, 5]. If PFR changes in AD relate to neurotransmitter status, then PFR testing may be useful as an objective, noninvasive monitor with which to follow disease progression and treatment efficacy.

As therapeutic trials in AD have shifted to earlier, preclinical intervention [33, 34], the need has grown for a practical screening test to identify those individuals on the pathway to symptomatic AD. Clinicopathologic studies at autopsy support the hypothesis of a protracted asymptomatic stage of AD, with the slow buildup of A*β* protein plaques beginning about 10–20 years prior to diagnosis [35–41]. PET A*β* neuroimaging provides a semiquantitative measure of NAB [16, 18–20]. However, while it is a valuable diagnostic and secondary screening biomarker, the procedure is not suitable as a primary screening technology for AD, due to cost, availability of PET scanners, invasiveness, and radiation dose. There is consequently a need for a noninvasive, cost-effective population-based AD screening technology to triage those requiring more extensive screening. Recent results from A*β* immunotherapy trials have shown promise, both for clearance of A*β* from the brain and for slowing cognitive decline in early or preclinical AD [33, 34], clearly underscoring the need for early detection.

To investigate pupillometry as a potential component of such an AD screening test, the present study investigated constriction phase pupil response in cognitively normal healthy control individuals stratified according to PET-determined NAB. The low-NAB group consists of cognitively and neuropathologically normal healthy control participants, while the high-NAB group consists of participants who have AD neuropathology but are still cognitively normal, suggesting they are in the preclinical phase of AD.

Since the cross-sectional data suggest a weaker pupil response in the high-NAB group, we hypothesized that longitudinal monitoring of pupil response may perform better at detecting preclinical AD. Natural variation in PFR between individuals may limit the utility of a single PFR test for AD screening; hence, it is possible that longitudinal monitoring might facilitate more accurate preclinical detection or monitoring of AD. Hence, we also investigated longitudinal changes in PFR over approximately 3 years. As the group means for each PFR parameter change were negative, the results suggested a decline in PFR over the period, consistent with the observed age-dependence of PFR parameters in the full cohort. The reduction in MCA was more pronounced in the high-NAB group, with a similar trend for MCV (not significant after multiple testing adjustment). Longitudinal change in MCA and MCV provided good classification accuracy (AUC 0.9); hence, pupillometric changes over time may have utility in detecting preclinical AD. The value of PFR testing may be in its use for providing a noninvasive monitor of physiological abnormality with which to follow disease progression and treatment efficacy.

Overall, the results add to the evidence of a weaker pupil flash response in AD and suggest that some PFR changes may occur in preclinical AD. To our knowledge, we are the only group to report on PFR differences with respect to NAB and preclinical AD. Cholinergic depletion may occur in preclinical AD, and pupillometry may have utility as a component of a practical screening test for early detection of AD. Additionally, longitudinal pupillometry could provide a practical monitoring test for disease progression or response to therapy.

The constriction phase of the PFR is primarily a parasympathetic response of the autonomic nervous system; hence, constriction PFR parameters can be used as an accurate method to assess the function of the neurotransmitter involved, acetylcholine [1, 3, 42]. Studies have suggested that PFR is sensitive to early cholinergic depletion which can lead to a decline in cognitive function. Cholinergic depletion may also occur in other diseases such as Parkinson's disease [43], which has also been reported to influence PFR [5, 9]. Hence, the specificity of the PFR changes in AD needs further investigation using cohorts which include individuals with other disorders that may affect the cholinergic system and PFR.

A major strength of this study is the well characterized cohorts, including the presence of neuroimaging data that enable deeper interrogation of associations between PFR parameters and AD. A limitation was the small number of participants (*N* = 27) in the longitudinal study; the results warrant further investigation with a similarly well-characterized, larger cohort.

## 5. Conclusions

This study demonstrates relationships between pupil response parameters, neocortical amyloid plaque load, and AD. Some PFR changes that are associated with diagnosed AD also occur in preclinical AD.

Pupillometry demonstrates potential as an adjunct (possibly together with blood or other biomarkers) (1) for accurate diagnosis of AD and monitoring of disease progress and response to therapy and (2) for low-cost and noninvasive detection of preclinical AD, recruitment into preclinical AD therapeutic trials and also monitoring response in these trials.

The results of this study suggest that PFR monitoring, rather than a single PFR test, might be more powerful as part of an early screening test for AD and for monitoring disease progress and response to intervention. Pupillometry is a low-cost, noninvasive technology that may reflect early cholinergic deficits preceding memory and cognitive decline.

## Figures and Tables

**Table 1:** Demographics and descriptive PFR analysis for HC and AD groups, with ANOVA, *χ*^2^ test, and GLM analysis.

	Healthy control	Alzheimer's disease	*p* value
Number of participants [*N*]	115	14	
Age: years [mean (±SD)]	72.9 (±5.3)	77.4 (±5.4)	**0.002** ^**~**^
Sex: male [*N* (%)]	56 (49)	10 (61)	0.11^†^
*APOE ε*4 carrier [*N* (%)]	27 (23)	12 (86)	**0.000001** ^†^
MCA [mm/sec^2^, mean (±SD)]	31.12 (±6.56)	26.84 (±4.23)	**0.030** ^‡^
MCV [mm/sec, mean (±SD)]	4.22 (±0.65)	3.41 (±0.55)	**0.00045** ^‡^
CV [mm/sec, mean (±SD)]	3.02 (±0.49)	2.53 (±0.44)	**0.0015** ^‡^
AMP [mm, mean (±SD)]	1.46 (±0.28)	1.15 (±0.28)	**0.0030** ^‡^

^~^Analysis of variance (ANOVA) for the continuous age demographic variable (*p* < 0.05 considered significant). ^†^*χ*^2^ test for categorical demographic variables (gender and *APOE ε*4 carrier status) (*p* < 0.05 considered significant). ^‡^*p* value from generalised linear model analysis of differences between groups (including significant confounders). Bold values significant after adjustment for false discovery rate (FDR) using the Benjamini and Hochberg method. *APOE ε*4 carrier status refers to carrier/noncarrier of an apolipoprotein E *ε*4 allele. SD refers to standard deviation, mm refers to millimetres, sec refers to seconds, PFR refers to pupil flash response, HC refers to healthy control, AD refers to Alzheimer's disease, GLM refers to generalised linear methods, MCA refers to maximum constriction acceleration, MCV refers to maximum constriction velocity, CV refers to average constriction velocity, and AMP refers to constriction amplitude.

**Table 2:** Demographics and descriptive PFR analysis for the HC group stratified according to neocortical amyloid burden (NAB), with ANOVA, *χ*^2^ test, and GLM analysis.

	Healthy control [low NAB]	Healthy control [high NAB]	*p* value
Number of participants [*N*]	77	38	
Age: years [mean (±SD)]	72.3 (±5.2)	74.0 (±5.3)	0.05734^~^
Sex: male [*N* (%)]	35 (49)	20 (53)	0.433^†^
*APOE ε*4 carrier: [*N* (%)]	10 (13)	17 (45)	**0.000202** ^†^
MCA [mm/sec^2^, mean (±SD)]	32.97 (±5.96)	30.08 (±7.2)	0.067^‡^
MCV [mm/sec, mean (±SD)]	4.48 (±0.63)	4.05 (±0.62)	**0.021** ^‡^
CV [mm/sec, mean (±SD)]	3.23 (±0.47)	2.92 (±0.42)	0.12^‡^
AMP [mean (±SD)]	1.54 (±0.29)	1.41 (±0.26)	0.77^‡^

^~^Analysis of variance (ANOVA) for the continuous age demographic variable (*p* < 0.05 considered significant). ^†^*χ*^2^ test for categorical demographic variables (gender and *APOE ε*4 carrier status) (*p* < 0.05 considered significant). ^‡^*p* value from the generalised linear model analysis of differences between groups (including significant confounders). Bold values significant after adjustment for false discovery rate (FDR) using the Benjamini and Hochberg method. *APOE ε*4 carrier status refers to carrier/noncarrier of an apolipoprotein E *ε*4 allele. NAB refers to neocortical amyloid burden, SD refers to standard deviation, mm refers to millimetres, sec refers to seconds, PFR refers to pupil flash response, HC refers to healthy control, AD refers to Alzheimer's disease, GLM refers to generalised linear methods, MCA refers to maximum constriction acceleration, MCV refers to maximum constriction velocity, CV refers to average constriction velocity, and AMP refers to constriction amplitude.

**Table 3:** Demographics and descriptive PFR analysis for the longitudinal HC group stratified according to neocortical amyloid burden (NAB), with ANOVA, *χ*^2^ test, and GLM and ROC analyses.

	Healthy control [low NAB]	Healthy control [high NAB]	*p* value
Number of participants [*N*]	19	11	
Age: years [mean (±SD)]	72.2 (±0.31)	72.1 (±4.3)	0.97^~^
Sex: male [*N* (%)]	7 (37)	8 (73)	**0.00026** ^†^
*APOE ε*4 carrier [*N* (%)]	4 (21)	10 (91)	**0.035** ^†^
Change in MCA [mm/sec^2^, mean (±SD)]	−1.49 (±1.80)	−5.66 (±3.10)	**0.0068** ^‡^
Change in MCV [mm/sec, mean (±SD)]	−0.19 (±0.17)	−0.55 (±0.42)	0.047^‡^
Change in CV [mm/sec, mean (±SD)]	−0.52 (±0.75)	−0.21 (±0.1)	0.62^‡^
Change in AMP [mm, mean (±SD)]	−0.24 (±0.32)	−0.13 (±0.09)	0.24^‡^

^~^Analysis of variance (ANOVA) for the continuous age demographic variable (*p* < 0.05 considered significant). ^†^*χ*^2^ test for categorical demographic variables (gender and *APOE ε*4 carrier status) (*p* < 0.05 considered significant). ^‡^*p* value from the generalised linear model analysis of differences between groups (including significant confounders). Bold values significant after adjustment for false discovery rate (FDR) using the Benjamini and Hochberg method. *APOE ε*4 carrier status refers to carrier/noncarrier of an apolipoprotein E *ε*4 allele. NAB refers to neocortical amyloid burden, SD refers to standard deviation, mm refers to millimetres, sec refers to seconds, PFR refers to pupil flash response, HC refers to healthy control, AD refers to Alzheimer's disease, GLM refers to generalised linear methods, MCA refers to maximum constriction acceleration, MCV refers to maximum constriction velocity, CV refers to average constriction velocity, and AMP refers to constriction amplitude.
